# Secondary motor areas for response inhibition: an epicortical recording and stimulation study

**DOI:** 10.1093/braincomms/fcac204

**Published:** 2022-08-04

**Authors:** Hirofumi Takeyama, Riki Matsumoto, Kiyohide Usami, Takuro Nakae, Akihiro Shimotake, Takayuki Kikuchi, Kazumichi Yoshida, Takeharu Kunieda, Susumu Miyamoto, Ryosuke Takahashi, Akio Ikeda

**Affiliations:** Department of Neurology, Japanese Red Cross Otsu hospital, Otsu 520-0046, Japan; Department of Neurology, Kyoto University, Kyoto 606-8507, Japan; Division of Neurology, Kobe University Graduate School of Medicine, Kobe 650-0017, Japan; Department of Epilepsy, Movement Disorders and Physiology, Kyoto University, Kyoto 606-8507, Japan; Department of Neurosurgery, Shiga Medical Center for Adults, Moriyama 524-8524, Japan; Department of Epilepsy, Movement Disorders and Physiology, Kyoto University, Kyoto 606-8507, Japan; Department of Neurosurgery, Kyoto University, Kyoto 606-8507, Japan; Department of Neurosurgery, Kyoto University, Kyoto 606-8507, Japan; Department of Neurosurgery, Ehime University, Touon 791-0295, Japan; Department of Neurosurgery, Kyoto University, Kyoto 606-8507, Japan; Department of Neurology, Kyoto University, Kyoto 606-8507, Japan; Department of Epilepsy, Movement Disorders and Physiology, Kyoto University, Kyoto 606-8507, Japan

**Keywords:** response inhibition, cortical stimulation, secondary motor areas, go/No-Go task

## Abstract

The areas that directly inhibit motor responses in the human brain remain not fully clarified, although the pre-supplementary motor area and lateral premotor areas have been implicated. The objective of the present study was to delineate the critical areas for response inhibition and the associated functional organization of the executive action control system in the frontal lobe. The subjects were eight intractable focal epilepsy patients with chronic subdural or depth electrode implantation for presurgical evaluation covering the frontal lobe (five for left hemisphere, three for right). We recorded event-related potentials to a Go/No-Go task. We then applied a brief 50 Hz electrical stimulation to investigate the effect of the intervention on the task. Brief stimulation was given to the cortical areas generating discrete event-related potentials specific for the No-Go trials (1–3 stimulation sites/patient, a total of 12 stimulation sites). We compared the locations of event-related potentials with the results of electrical cortical stimulation for clinical mapping. We also compared the behavioural changes induced by another brief stimulation with electrical cortical stimulation mapping. As the results, anatomically, No-Go-specific event-related potentials with relatively high amplitude, named ‘large No-Go event-related potentials’, were observed predominantly in the secondary motor areas, made up of the supplementary motor area proper, the pre-supplementary motor area, and the lateral premotor areas. Functionally, large No-Go event-related potentials in the frontal lobe were located at or around the negative motor areas or language-related areas. Brief stimulation prolonged Go reaction time at most stimulation sites (66.7%) [*P* < 0.0001, effect size (d) = 0.30, Wilcoxon rank sum test], and increased No-Go error at some stimulation sites (25.0%: left posterior middle frontal gyrus and left pre-supplementary motor area). The stimulation sites we adopted for brief stimulation were most frequently labelled ‘negative motor area’ (63.6%), followed by ‘language-related area’ (18.2%) by the electrical cortical stimulation mapping. The stimulation sites where the brief stimulation increased No-Go errors tended to be labelled ‘language-related area’ more frequently than ‘negative motor area’ [*P* = 0.0833, Fisher’s exact test (two-sided)] and were located more anteriorly than were those without a No-Go error increase. By integrating the methods of different modality, namely, event-related potentials combined with brief stimulation and clinical electrical cortical stimulation mapping, we conducted a novel neuroscientific approach, providing direct evidence that secondary motor areas, especially the pre-supplementary motor area and posterior middle frontal gyrus, play an important role in response inhibition.

## Teaser

By a novel neuroscientific approach integrating event-related potentials, brief stimulation, and clinical electrical cortical stimulation mapping, Takeyama *et al*. report that the secondary motor areas, especially the pre-supplementary motor area and the posterior middle frontal gyrus, have an important role in response inhibition.

## Graphical Abstract

**Graphical Abstract:**
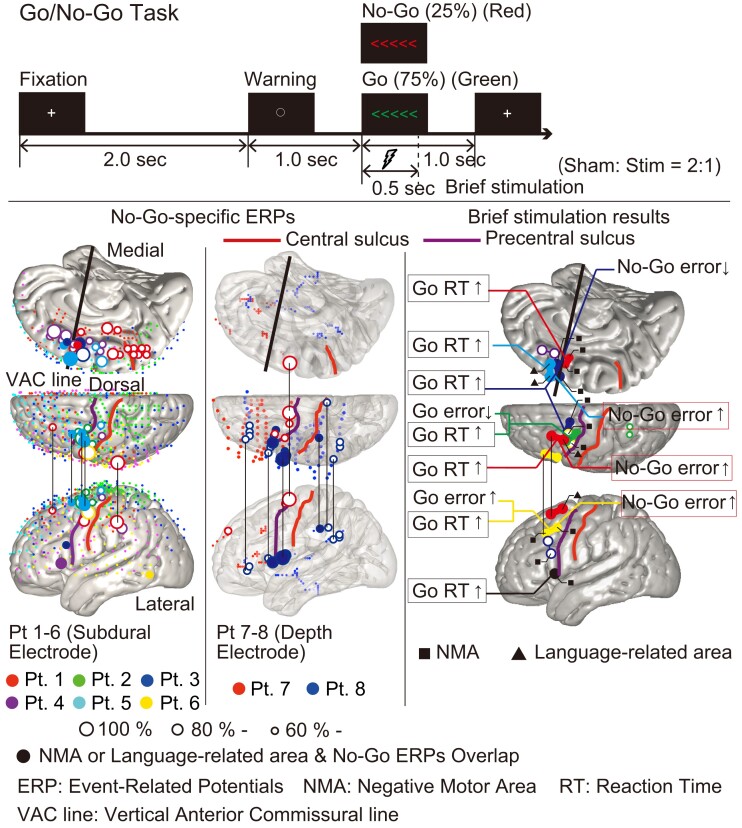

## Introduction

Response inhibition is an important executive function, which cancels an initiated response; it suppresses responses that are no longer required or are inappropriate. Response inhibition is impaired in various neurological diseases, such as Parkinson’s disease, leading to impulsivity, which compromises the patient’s quality of life.^1^

The Go/No-Go task is commonly used for the assessment of response inhibition.^2–6^ In the Go/No-Go task, a subject is instructed to respond to a ‘Go’ signal and to withhold their response to a ‘No-Go’ signal. A meta-analysis of 30 Go/No-Go functional MRI (fMRI) studies in humans showed that the right pre-supplementary motor area (pre-SMA), inferior frontal gyrus, and dorsolateral prefrontal cortex play an important role in response inhibition.^7^ In addition, human transcranial magnetic stimulation (TMS) studies have shown the importance of the pre-SMA, right inferior frontal gyrus and left dorsal premotor area (PMd).^8,9^

TMS studies cannot record neural activity, and fMRI studies can only indirectly evaluate neural activity from the correlational haemodynamic responses. On the other hand, electrocorticography (ECoG) and direct electrical cortical stimulation (ECS) using intracranial electrodes can directly record the electrophysiological cortical neural activity and delineate the cortical areas necessary for a particular function by producing transient functional disturbances, respectively. For example, the previous ECoG studies without ECS showed that the lateral premotor areas (the middle or inferior frontal gyrus) play an important role in response inhibition.^10,11^ A combination of ECoG and ECS can provide more direct evidence of the functional localization of response inhibition than can fMRI or TMS studies.^12^

The cortical regions that are critical for response inhibition have also been considered vital for other higher brain functions.^13^ For example, ‘negative motor areas (NMAs),’ in which ECS causes inhibition or cessation of ongoing movement,^14^ have been found in the pre-SMA, inferior frontal gyrus and premotor areas.^15^ ECS of the human pre-SMA evoked speech arrest or a slowing of speech.^16,17^ Another study demonstrated that the same neurons in the pre-SMA are involved in both task switching and response inhibition (or facilitation) during the Go/No-Go task in monkeys.^18^ Therefore, for a comprehensive understanding of the functional localization of response inhibition, it is important to reveal the functional overlap between response inhibition and other higher brain functions.

The objective of the present study was to delineate the critical areas for response inhibition and the associated functional organization of the human executive action control system. For this purpose, we analyzed the clinical ECS mapping results, event-related potentials (ERPs) to the Go/No-Go task, the behavioural changes induced by ECS during the Go/No-Go task. To the best of our knowledge, no other study has attempted direct ECS during a cognitive task for the assessment of response inhibition in human subjects.

## Materials and methods

### Subjects

The subjects were eight patients (four males) with medically intractable focal epilepsy who were treated in our hospital with intracranial electrode implantation in the lateral and/or medial frontal area for presurgical evaluation from 2013 through 2017. Six patients had subdural electrode implantation, and the other two had depth electrode implantation for stereo EEG. Five patients had left hemisphere coverage, whereas three had right hemisphere coverage. Five patients (patient 1, 2, 4–6) had electrode implantation on the language-dominant hemisphere, which was decided by the pre-operative intracarotid propofol procedure.^19^ The demographics of the patients are shown in Supplementary Table 1. All patients provided written informed consent. The protocol used was in accordance with the Declaration of Helsinki and was approved by the ethics committee of Kyoto University graduate school and faculty of medicine (No. C533).

### Electrode placement and localization

Electrode locations are shown in Supplementary Fig. 1. The implanted electrodes were the subdural electrodes (platinum-made, inter-electrode distance of 1 cm, recording surface diameter of 2.3 mm, AD-TECH, WI, USA) for patients 1–6, and depth electrodes (platinum-made, inter-electrode distance: 5, 6, or 10 mm, diameter: 1.12 mm, AD-TECH, WI, USA) for patients 7 and 8. After excluding electrodes inappropriate for the analysis due to poor recording conditions caused by disconnection of the electrode wire or floating of the electrode from the brain surface, the number of electrodes in total was 695 and the number per patient ranged from 60 to 104 [86.9 ± 16.2, mean ± standard deviation (SD)].

The methods of standard electrode placement and co-registration to the Montreal Neurological Institute (MNI) standard space are described in detail elsewhere.^20,21^ In short, magnetization-prepared rapid gradient-echo sequences were obtained as anatomical T_1_-weighted volume data before and after electrode implantation. We determined the electrode coordinates in the image taken after implantation based on the hypointense signal caused by the electrode’s platinum alloy properties. Next, we co-registered these coordinates for each patient non-linearly to the scan image obtained before implantation and mapped this to the MNI standard space (ICBM-152) using FMRIB's non-linear image registration tool (www.fmrib.ox.ac.uk/fsl/fnirt).

### Behavioural paradigm

**Figure 1:**
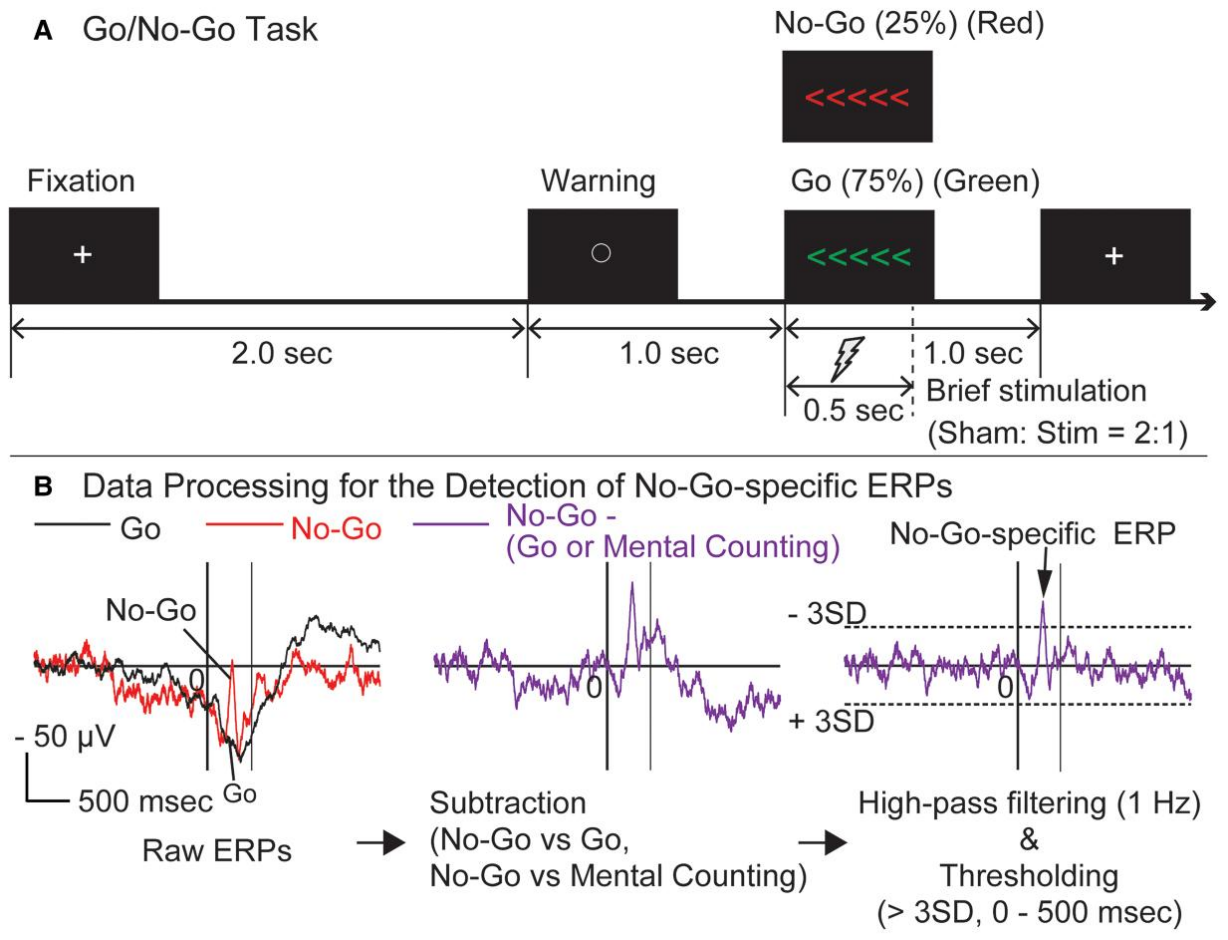
**The Go/No-Go task and data analysis.** (**A**) The Go/No-Go task. One trial consisted of the presentation of the fixation point (2.0 s), warning signal (1.0 s) and Go or No-Go signal (1.0 s). One session consisted of 48 trials. Each Go/No-Go signal was presented pseudo-randomly with a fixed probability (Go: 75% = 36 trials/1 session, No-Go: 25% = 12 trials/1 session). For the intervention during the Go/No-Go task, direct ECS (50 Hz, pulse width of 0.3 ms, alternating polarity, 4–8 mA) was delivered at the onset of the display of a Go or No-Go signal for 0.5 s. The ratio of sham and stimulation trials was 2:1 in both the Go and No-Go trials (within-session pseudo-randomization). (**B**) Data processing for the detection of No-Go-specific ERPs. Two patterns of subtraction (1: No-Go versus Go trials of the Go/No-Go task, 2: No-Go trials of the Go/No-Go task versus No-Go trials of the mental counting task) were applied on the raw ERP data. The No-Go-specific ERPs were then extracted using high-pass filtering (above 1 Hz) and thresholding (above 3 SD of baseline activity during the time window between 0 to +500 ms) for each subtracted data.

We used the Go/No-Go task to evaluate the response inhibition function (Fig. 1A). A Go signal or a No-Go signal was presented pseudo-randomly with a fixed probability (Go: 75%, No-Go: 25%). One session consisted of 48 trials (36 Go and 12 No-Go signals). Both the Go and No-Go signals were shown as five arrowheads lined up horizontally, and the only difference between them was in the colour of the arrowheads (Go signal: green, No-Go signal: red). When a Go signal appeared, the patient was asked to press the ‘left’ or ‘right’ button according to the direction of the arrows as quickly as possible with the index finger of the hand contralateral to the side of the electrode implantation. On the other hand, when a No-Go signal was presented, the patient had to withhold pressing the button. The visual angle of the Go/No-Go signal (five arrows) was 3.91 wide and 0.651 tall. The direction of the arrowheads in Go/No-Go signals was fixed as rightward or leftward throughout each session (rightward direction in half of the sessions), and the patients were informed of the direction before the start of each session. The patient watched an liquid-crystal display screen at a distance of 1.0 m, sitting comfortably in Fowler’s position on the bed. In each trial, an open circle was presented in the centre of the screen for 1 s, and then it was replaced by the Go or No-Go signal for 1 s (Fig. 1A). The patient was instructed to look at the cross in the centre of the display during the 2 s inter-trial interval, and not to blink while the circle or the Go/No-Go signal appeared. We confirmed that the patient could perform the task appropriately in the rehearsal before the implantation surgery. The Go/No-Go task was performed twice on separate days: first for ERP recording and second for the brief stimulation.

In addition, we employed the ‘mental counting task’ as a control task to differentiate the brain activity for response inhibition from that for signal perception, since the mental counting task, with the usage of the same visual stimuli as the Go/No-Go task, demands a subject to pay attention to No-Go signal without any motor response. In the mental counting task, the patient was instructed to mentally count the number of No-Go signals without any motor or vocal response. The patient held the button-pressing apparatus in the hand during both the mental counting task and the Go/No-Go task to standardize the patient’s physical condition between the two tasks. All the signals used in the mental counting task were the same as in the Go/No-Go task, the only difference being the type of response of the patients. In the mental counting task, one session consisted of 48 trials, and the number of No-Go trials was pseudo-randomized among the sessions, in average 12 No-Go trials (9–14 trials). To maintain the attention of the patients, we interviewed them after each session how many No-Go signals they had counted mentally.

### Event-related potential analysis

We averaged the ECoGs time-locked to the onset of the Go or No-Go signal to obtain the ERPs by off-line manner (MATLAB scripts, custom-made in Matlab version 2019b). Averaging was performed separately for (i) Go trials in a Go/No-Go task, (ii) No-Go trials in a Go/No-Go task and (iii) No-Go trials in the mental counting task. We set a total of 4 s as the time window (–2 to +2 s from the onset of the Go/No-Go signal). The initial 1 s of the analysis window was used as the baseline for averaging.

By carefully monitoring the ECoG throughout the study, we confirmed that no seizures had occurred. The ECoG, electro-oculogram and all related signals, such as the timing of visual stimuli and button-press responses, were digitally recorded and stored on the hard disk in the recording system (EEG1100/1200, Nihon Koden, Tokyo, Japan). The data were sampled at 1000 or 2000 Hz with a band-pass filter of 0.016–300 Hz or 0.016–600 Hz, respectively. The signals recorded by the electrodes were referenced to a scalp electrode placed on skin of the mastoid process contralateral to the side of electrode implantation. The data were manually inspected for the presence of interictal discharges, 60 Hz noise contamination, and other noises to decrease signal-to-noise ratio. Epochs containing clinical or subclinical EEG seizure patterns were excluded for all electrodes in the dataset. If eye blinks occurred between –50 ms to +50 ms from the onset of the Go or No-Go signal presentation, such a trial was excluded from further analysis, as described previously.^22^ We analyzed only the successful Go and No-Go trials for ERPs.

Based on preliminary visual inspection, we focused on the ERP components in early peak latency (< 500 ms) containing outstanding apical waveforms in the No-Go trials. Thus, we performed data processing for the detection of No-Go-specific ERPs (Fig. 1B). First, we performed two subtraction processes separately: (i) subtraction of the Go ERPs from the No-Go ERPs for the Go/No-Go task and (ii) subtraction of the No-Go ERPs of the mental counting task from the No-Go ERPs of the Go/No-Go task. Second, each difference ERP was high-pass filtered at 1 Hz to focus on the apical components of the ERPs. Then the filtered data were thresholded within the time window between 0 and 500 ms at 3 SDs of the ERP amplitude in the baseline window (–2 to –1 s from the onset of the Go/No-Go signal presentation). Finally, we defined the No-Go-specific ERPs as the ERPs that remained after the 3-SD thresholding in both sets of subtracted data. The amplitude of each No-Go-specific ERP was normalized as the percentage (%) of that of the maximum response in each patient. For the display purpose to show the core regions of response inhibition in the figures, we selected electrodes with No-Go-specific ERPs (‘large No-Go ERPs’) with an amplitude higher than 60% of the maximum (60––100%) No-Go-specific signal and plotted the anatomical locations of the selected electrodes for all patients in the MNI standard space.

### Brief stimulation study

The stimulation site for the brief stimulation was determined based on previous studies on response inhibition.^7^ In the medial frontal area, we selected for stimulation the electrodes anterior to the vertical anterior commissure (VAC) line, namely, the pre-SMA. In the lateral frontal area, we selected the premotor areas anterior to the precentral sulcus, namely, the PMd and ventral premotor area (PMv), as the stimulation sites. We defined the vertical boundary between the PMd and PMv as the midline of the middle frontal gyrus.^23,24^ In each patient, we selected 1–3 stimulation sites (i.e. pairs of adjacent electrodes) for brief stimulation among candidate electrodes in frontal areas showing discrete ERPs in No-Go trials. This selection was based on the regions relevant to response inhibition identified in previous studies, namely, the pre-SMA, PMd or PMv, except for one parietal stimulation site in Patient 2. Owing to the clinical limitation, time constraints between ERP recording and brief stimulation study made it difficult for us to interpret the results of No-Go-specific ERPs and to choose the stimulation sites. Thus, by visual inspection, we identified the discrete No-Go ERPs, which were the relatively salient ERPs between 0 to +500 ms in the No-Go trials of the Go/No-Go task, compared with the Go trials in the Go/No-Go task and the No-Go trials in the mental counting task. We retrospectively confirmed that the stimulation sites successfully recorded the No-Go-specific ERPs according to the stringent criteria described previously, except for one stimulation site in Patient 1 (PMd) and Patient 2 [superior parietal lobule (SPL)] as follows. Namely, No-Go-specific ERPs were recorded at the electrode (E09) adjacent to the stimulation site (E07-E08) in Patient 1, whereas no No-Go-specific ERPs were recorded either at the stimulation site or its adjacent electrode in Patient 2. The number of stimulation sites in each patient was decided depending on clinical limitation for this study.

To fully evaluate the behavioural changes during brief stimulation, we adjusted electrical stimuli to be shorter and smaller than those used for clinical ECS mapping so that the brief stimulation did not produce any afterdischarges or positive or negative motor responses as reported elsewhere.^22,25^ A 50 Hz stimulation (monophasic rectangular pulse, pulse width of 0.3 ms, alternating polarity) was delivered via two adjacent electrodes (stimulation electrode pair) at 4–8 mA for 0.5 s from the onset of the signal presentation in the Go or No-Go trials (Fig. 1A), using a constant-current stimulator (Electrical stimulator SEN-7203, Nihon Koden, Tokyo, Japan). We performed six sessions of the Go/No-Go task at each stimulation site. Each session consisted of 48 trials (36 Go and 12 No-Go signals). We employed a within-block control (sham stimulation) to analyze the effect of the brief stimulation. The ratio of sham and stimulation trials was 2:1 in both the Go and No-Go trials (Go: 24 sham/12 stimulation trials; No-Go: 8 sham/4 stimulation trials in each session). The order of the sham and stimulation trials was pseudo-randomized within each session. We generated the sounds of the relay switch needed for stimulus delivery in sham trials so that the subject could not differentiate between the real and sham stimulation trials. By carefully monitoring the ECoG, we confirmed that no seizure or afterdischarge occurred during the brief stimulation. At the end of each session, we asked the patient to report symptoms or unusual feelings during the task, if any, to exclude the existence of stimulus-related symptoms.

### Behaviour analysis

We compared the number of errors in the Go/No-Go trials and the reaction time (RT) of the Go trials in the sham trials with that in the stimulation trials to test the hypothesis that direct ECS impairs task performance. We defined the error in the No-Go trial as pressing the button on the No-Go signal, the error in the Go trial as not pressing the button on the Go signal, and RT as the time between the onset of a Go signal and the timing of a button press. Z-score normalization was applied to the RT data, based on all RT data of each patient in the brief stimulation study. Then, we compared all patients’ z-scores between the sham and stimulation trials.

### Electrical cortical stimulation mapping

ECS mapping was performed as a part of the clinical presurgical evaluation. Repetitive square-wave currents of alternating polarity with a pulse width of 0.3 ms and a frequency of 50 Hz were delivered for 1–5 s to the cortex through a pair of adjacent electrodes. The current intensity was increased gradually from 1 to 15 mA until positive motor responses, for example, muscle twitch or tonic posturing, appeared. We evaluated only the trials without after discharges. In the absence of a positive response, the patient was asked to perform rapid alternating movements of the tongue, hands, and feet during cortical stimulation (10–15 mA, 5 s). Once the patient was unable to continue these movements, that is, when a negative motor response was elicited, we labelled the stimulation area as the ‘NMA’. The stimulation method has been reported elsewhere.^24^

When neither positive nor negative motor responses were elicited in an area by the stimulation, we further examined the effect of the stimulation on language function. The language tasks used in our institute consisted of overt sentence reading, picture naming, verbal command, picture-word matching, ‘kanji’ reading, ‘kana’ reading, and repetition of meaningless words.^25^ ‘Kanji’ and ‘kana’ are different types of Japanese script. The deficit induced by the electrical stimulation was categorized as ‘mistake,’ ‘slowing,’ or ‘arrest’ by reaching an agreement among the examiners including at least one board-certified neurologist. If a deficit in any language task was observed, we defined the cortical area under stimulation as a ‘language-related area.’

We plotted the coordinates of the NMA and language-related areas for all patients in the MNI standard space for comparison with the locations of the No-Go-specific ERPs because we hypothesized that NMA and language-related areas anatomically overlap with the response inhibition areas in the frontal lobe, based on previous studies^15–17^ and preliminary inspection of the ECS mapping results. We excluded electrodes with inconclusive or no ECS mapping results from further analysis. We identified electrodes that showed an overlap of the large No-Go ERP area with either NMAs or language-related areas and calculated their ratios (%) relative to all implanted electrodes (‘positive rate’) in each patient. In addition, we compared the results of the brief stimulation with the ECS mapping results.

### Statistical analysis

The χ^2^ test, Wilcoxon rank sum test and Fisher’s exact test (two-sided) were applied for the evaluation of errors, RT and positive rate and A–P axis, functional overlaps, respectively (*P*-value threshold: 0.05). As described in ‘Results’ section, statistical analysis was applied on a single patient (errors, RT), or on all patients as a whole (A–P axis, functional overlaps).

### Data availability

The data are not publicly available due to privacy or ethical restrictions.

## Results

### Event-related potential

For ERP recording, a total of eight sessions of the Go/No-Go and mental counting tasks were performed by all patients except for Patient 5, who could complete only five sessions of the Go/No-Go task and three sessions of the mental counting task due to time constraints. Both tasks were performed on the same day by all patients except for Patients 1, 2 and 4, who accomplished each task independently on separate days due to time constraints. Since eye blinks occurred from –50 to +50 ms from the onset of the signal presentation, we excluded 10 Go trials and 6 No-Go trials of the Go/No-Go task in Patient 6, and 1 No-Go trial of the mental counting task in Patient 2 from further analysis.

The average RT of successful Go trials was 365.3 ms, and the average error rates of Go and No-Go trials were 2.2% and 5.8%, respectively (Supplementary Table 2).

**Figure 2:**
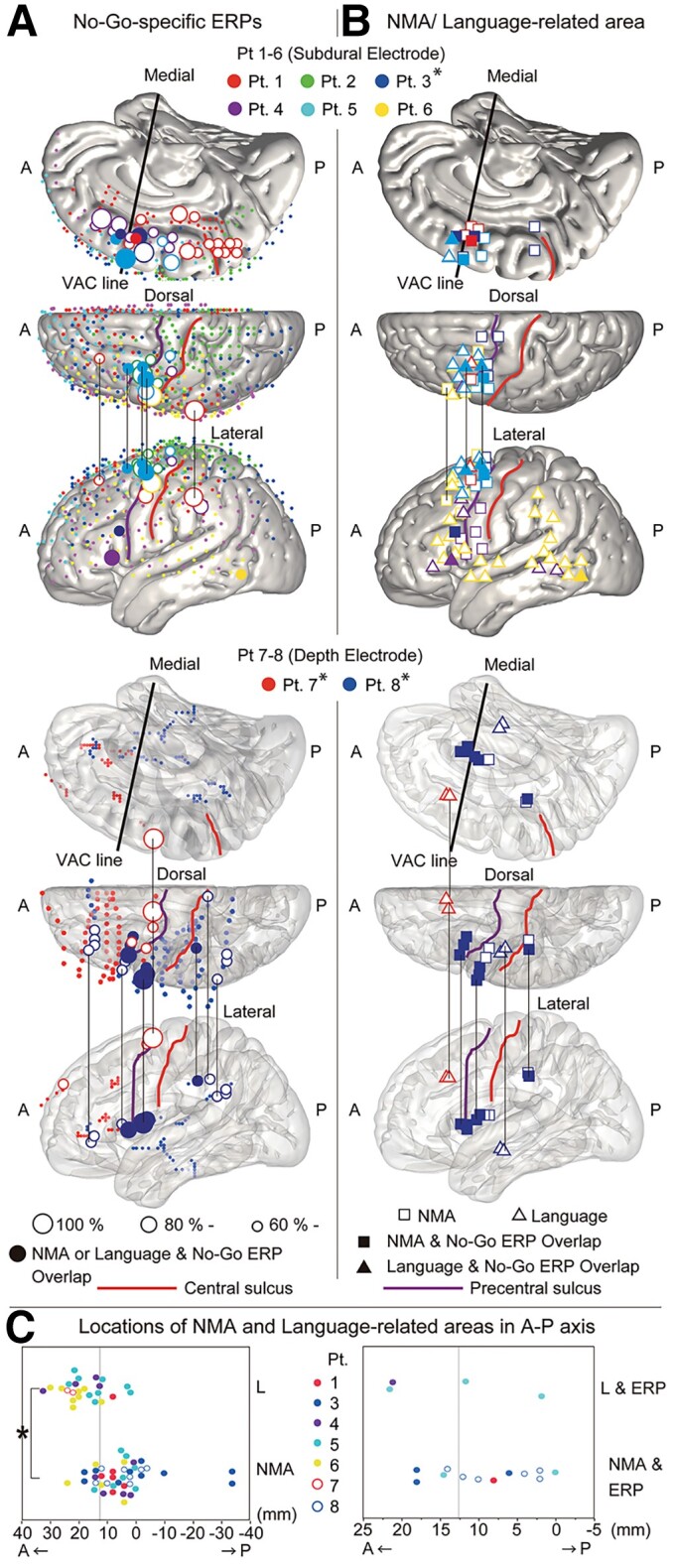
**No-Go-specific ERPs and functional overlap.** (**A**) Results of No-Go-specific ERP assessment in patients 1–6 (with subdural electrode implantation) (top three panels) and patient 7 and 8 (lower three panels) (with depth electrode implantation). The locations of the electrodes that recorded the large No-Go ERPs are shown as large circles. The circles indicate the relative amplitude of the No-Go-specific ERPs within each patient [large size: 100% (maximum), middle size: ≥ 80%, small size: ≥ 60%]. The filled circles represent the electrodes that overlapped with NMA or language-related areas as identified using ECS mapping. The small dots indicate the locations of the implanted electrodes where the high amplitude No-Go-specific ERPs (≥60%) were absent. (**B**) Functional overlap between response inhibition and other brain functions. The figures show the location of the electrodes where NMAs or language-related areas were identified using ECS mapping. The squares represent the NMAs, whereas the triangles show the language-related areas. The filled squares and triangles show the stimulation sites that overlapped with large No-Go ERP sites. The central sulcus, precentral sulcus and VAC lines are shown as the thick lines. * The electrodes in patients 3, 7 and 8 are swapped from the right to left side for display purposes. (**C**) Locations of the NMAs and language-related areas in the A–P axis. The vertical lines in the figures show the coordinate of the intersection point of precentral sulcus and inferior frontal sulcus in the A–P axis. Left panel: the locations of the NMAs (*n* = 40) and language-related areas (*n* = 24) (all patients, only frontal lobe electrodes) along the A–P axis. The locations of the language-related areas were more anterior than those of NMAs. *P < 0.0001 (Wilcoxon rank sum test). Right panel: the locations of the electrode with the overlapping results [NMA with large No-Go ERP (*n* = 12), language-related area with large No-Go ERP (*n* = 4)] along the A–P axis. Note that we could not perform the statistical test due to the small sample size for the data shown in the right panel. L, language-related area; NMA, negative motor area.

We plotted the anatomical locations of the electrodes that recorded large No-Go ERPs (No-Go-specific ERPs with amplitude ≥ 60% in each patient) in the MNI standard space (Fig. 2A). Large No-Go ERPs were recorded broadly in both the medial and lateral regions of the frontal and parietal lobes. In the areas anterior to the primary motor area, large No-Go ERPs were observed predominantly in the secondary motor areas, made up of the SMA proper, the pre-SMA, and the lateral premotor areas.^26^ Large No-Go ERPs were also observed in the dorsal and ventral parts of the precentral gyrus, supramarginal gyrus, paracentral lobule and posterior cingulate gyrus. We could not find clear laterality in the distribution of the large No-Go ERPs by visual inspection, as analysis of laterality was difficult due to the small number of patients with right hemisphere coverage (*n* = 3).

### Brief stimulation results

**Figure 3:**
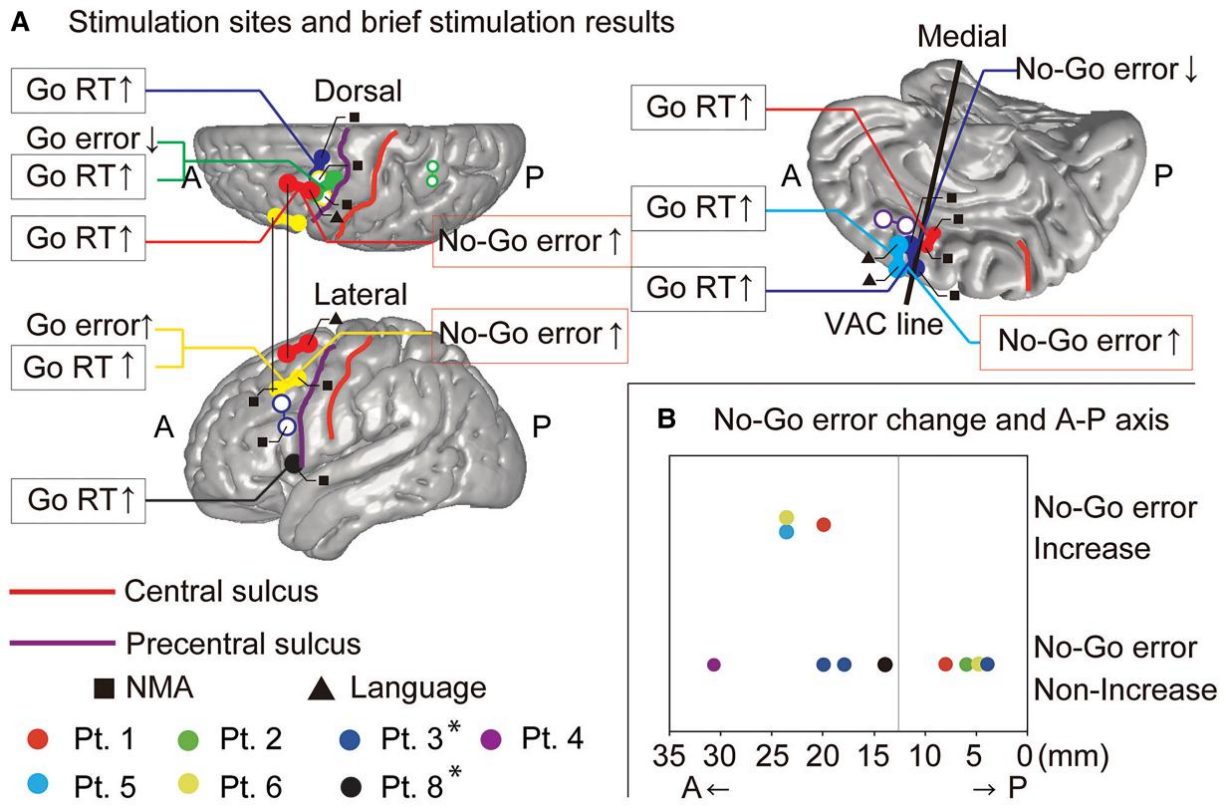
**The anatomical locations of stimulation sites and the results of brief stimulation.** (**A**) Upper left, lower left and right panels: the three-dimensional brain viewed from the dorsal, lateral, and medial sides, respectively. The central sulcus, precentral sulcus, and VAC lines are shown as the thick lines. The stimulation sites where brief stimulation led to a significant prolongation of the response time in Go trials and a significant change in the error rate are indicated. Go RT prolongation and No-Go error increase are highlighted by the boxes. The open circles represent the stimulation electrode sites where the brief stimulation did not result in a significant behavioural change. The stimulation electrode sites that were labelled as NMA or language-related areas in ECS mapping are annotated by filled squares or triangles, respectively. *The electrodes in patients 3 and 8 are swapped from the right to left side for display purposes. (**B**) The Y-coordinates of the stimulation sites along the A–P axis with the presence (*n* = 3) or absence (*n* = 8) (all patients, only frontal lobe electrodes) of increased No-Go error after stimulation were compared. The vertical lines in the figures show the coordinate of the intersection point of precentral sulcus and inferior frontal sulcus in the A–P axis. Note that we could not perform the statistical test because of the small sample size.

**Figure 4:**
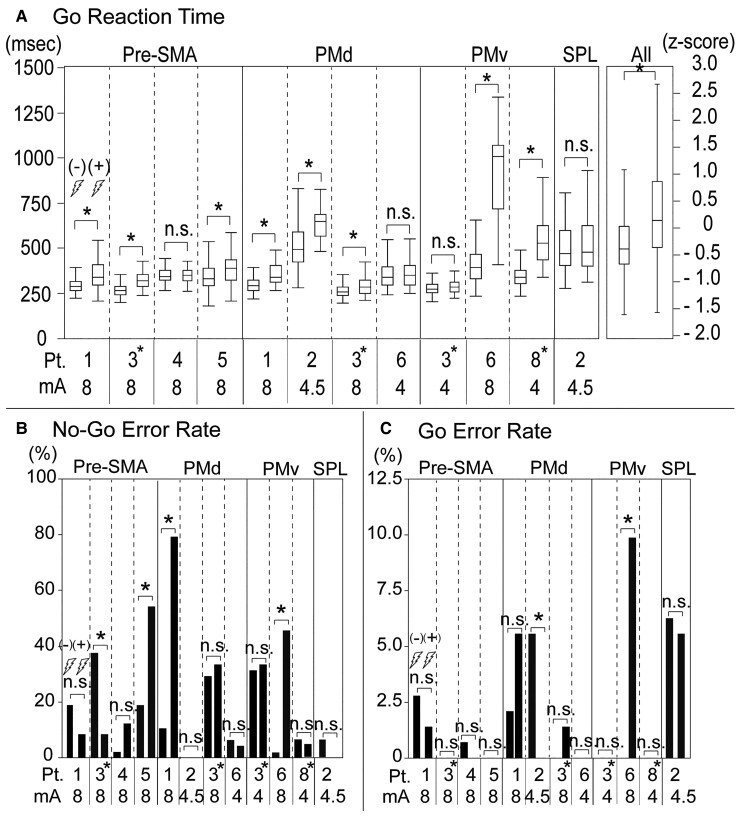
**Behavioural changes due to brief stimulation during the Go/No-Go task.** (**A**) Changes in reaction time in the Go trials. Sham Go trials: *n* = 144, stimulation Go trials: *n* = 72, in each patient. *P < 0.05 Wilcoxon rank sum test. n.s., not significant. (**B**) Changes in the error rate in the No-Go trials. Sham No-Go trials: *n* = 48, stimulation No-Go trials: *n* = 24, in each patient. *P < 0.05 χ^2^ test. (**C**) Changes in the error rate in the Go trials. Sham Go trials: *n* = 144, stimulation Go trials: *n* = 72, in each patient. *P < 0.05 χ^2^ test. The left (right) bar in each column denotes the results of the sham (real) stimulation. Pre-SMA, pre-supplementary motor area, PMd, dorsal premotor area, PMv, ventral premotor area, SPL, superior parietal lobule.

We performed the brief stimulation study in all patients except for Patient 7, who did not have time for the brief stimulation study due to the clinical examinations needed for epileptic surgery. The stimulation sites and brief stimulation results are summarized in Fig. 3A. Figure 4 shows the behavioural changes in each patient in detail. The brief stimulation was performed in the pre-SMA (left: three patients, right: one patient), PMd (left: three patients, right: one patient), PMv (left: one patient, right: two patients) and left SPL (one patient) (Fig. 3A). The stimulation intensity was lowered to 4.0 or 4.5 mA at some stimulation sites to avoid seizure induction (Patients 2 and 3) or the emergence of negative motor responses (Patients 6 and 8) (Table 1 and Fig. 4). None of the patients reported any positive symptoms or unusual feelings during the task.

**Table 1:** ECS mapping results in the stimulation sites for brief stimulation

Pt.	Stimulation site [electrode name, current intensity in brief stimulation (mA)]	ECS mapping results (deficit) [current intensity (mA), duration (s) in ECS mapping]
1	L Pre-SMA (F03-F04, 8 mA)	F03 & F04: **NMA** (Slowing of tongue, bilateral hands and feet) (10 mA, 5 s)
1	L PMd (E07-E08, 8 mA)	E07: **Language** (Arrest of all tasks) (14–15 mA, 5 s) E08: No function (15 mA, 4 s)
2	L PMd (B05-B10, 4.5 mA)	B05: Right face motor (7 mA, 0.5 s) B10: Seizure induction (5 mA, 1 s)
2	L SPL (F02-F10, 4.5 mA)	F02 & F10: No function (10 mA, 5 s)
3	R Pre-SMA (I06-I14, 8 mA)	I06: **NMA** (Slowing of L hand and tongue) (14–15 mA, 5 s) I14: **NMA** (Arrest of L hand and tongue & slowing of R hand and bilateral feet) (10 mA, 5 s)
3	R PMd (J05-J13, 8 mA)	J05: No function (15 mA, 5 s). J13: **NMA** (Slowing of L hand) (10 mA, 5 s)
3	R PMv (K01-K02, 4 mA)	K01: **NMA** (Arrest of bilateral hand and tongue) & Seizure induction (sensory aura) (8 mA, 5 s) K02: Seizure induction (sensory aura) (4–5 mA, 5 s)
4	L Pre-SMA (G14-G15, 8 mA)	Not evaluated
5	L Pre-SMA (E06-E14, 8 mA)	E06: **Language** (Arrest of all tasks) (9 mA, 5 s) E14: **Language** (Arrest of naming) (15 mA, 5 s)
6	L PMd (A15-A20, 4 mA)	A15 & A20: **NMA** (Arrest of R hand) (5 mA, 0.5 s)
6	L PMv (D04-D05, 8 mA)	D04 & D05: **NMA** (Slowing of R hand and tongue) (9 mA, 5 s)
8	R PMv (Q02-Q03, 4 mA)	Q02 & Q03: **NMA** (Arrest of L hand, slowing of R hand) (7 mA, 5 s)

L, left; R, right, RT, reaction time; NMA, negative motor area; Pre-SMA, pre-supplementary motor area; PMd, dorsal premotor area; PMv, ventral premotor area, SPL, superior parietal lobule.

In the majority (66.7%) of the stimulation sites, electrical stimulation prolonged the Go RT in the pre-SMA (left: two patients, right: one patient), PMd (left: two patients, right: one patient), and PMv (left: one patient, right: one patient) compared with the sham stimulation (*P* < 0.05, Wilcoxon rank sum test) (Figs. 3A and 4A). To evaluate the overall effects of stimulation on the Go RT regardless of the patient or stimulation site, we performed z-score normalization for each patient, and then combined the *z*-scores of all the patients. This analysis revealed significant Go RT prolongation by the electrical stimulation compared with that of sham stimulation [*P* < 0.0001, effect size (d) = 0.30, Wilcoxon rank sum test] (Fig. 4A, the rightmost column). In some stimulation sites (25.0%), we observed an increase in the No-Go errors, a behavioural index of the failure of response inhibition, in the left pre-SMA (one patient), left PMd (one patient) and left PMv (one patient) (*P* < 0.05, χ^2^ test) (Figs. 3A and 4B). As shown in Fig. 3A, in the lateral premotor areas, both the stimulation sites with the increased No-Go errors (red: PMd, yellow: PMv) were located on the same gyrus, namely the posterior part of the left middle frontal gyrus (pMFG). We also observed a decrease in No-Go errors in the right pre-SMA (one patient), Go errors increased in the left pMFG (1) and Go errors decreased in the left pMFG (1) (*P* < 0.05, χ^2^ test) (Figs. 3A and 4B, C).

### Comparison of event-related potential and the brief stimulation results with electrical cortical stimulation mapping

The NMAs and language-related areas were located at or around the neighbourhood of the large No-Go ERP sites (Fig. 2A and B). The NMAs and large No-Go ERP sites overlapped in the pre-SMA, the most anterior part of the SMA proper, the pMFG, the posterior inferior frontal gyrus, pars opercularis, the anterior insula and the inferior parietal lobule, while language-related areas and large No-Go ERP sites coexisted in the pre-SMA, pMFG, posterior inferior frontal gyrus, and posterior middle temporal gyrus (Fig. 2A and B). The positive rate (%) was not different between the NMA and language-related areas [NMA: 8.9 ± 5.9% (*n* = 8); language-related area: 8.9 ± 9.7% (*n* = 8), *P* = 0.7513, *d* = 0.01, Wilcoxon rank sum test], or between NMAs overlapping large No-Go ERP sites and language-related areas overlapping large No-Go ERP sites [NMA with large No-Go ERP: 3.1 ± 5.0% (*n* = 7); language-related area with large No-Go ERP: 1.4 ± 2.0% (*n* = 6), *P* = 0.6516, d = 0.87, Wilcoxon rank sum test] (Supplementary Fig. 2).

In the frontal lobe, 11 of 12 stimulation sites selected for brief stimulation were evaluated by ECS mapping for clinical necessity. Seven stimulation sites (63.6%) were labelled as NMA, whereas two sites in the left pMFG or left pre-SMA (18.2%) were labelled as language-related areas (Table 1). In the language-related areas, all language tasks were arrested during ECS mapping. Please see Table 1 for more information about the type of language deficit elicited by ECS. Brief stimulation at both the stimulation sites in the language-related area elicited an increase in No-Go errors, whereas only one of seven stimulation sites in the NMA increased the No-Go error. Because of the limited number of stimulation sites for brief stimulation, this finding did not reach statistical significance [*P* = 0.0833, Fisher’s exact test (two-sided)] (Table 2).

**Table 2:** Relationship between No-Go error increase and ECS mapping results in the stimulation sites for brief stimulation

	No-Go error increase (+)	No-Go error increase (–)	Total
NMA	1	6	7
Language-related area	2*	0	2
Total	3	6	9

* *P* = 0.0833 [Fisher’s exact test (two-sided)].

From these results, we hypothesized that the human executive control system in the secondary motor areas has functional differentiation along the anterior–posterior (A–P) axis. Therefore, we analyzed the results of the ERP, brief stimulation, and ECS mapping from the viewpoint of the electrode locations in the A–P axis (Y coordinate in the MNI standard space). We supposed that stimulation of the anterior cortical areas played an important role in the behavioural changes that were observed; thus, we analyzed the location of the more anteriorly located stimulation electrodes in the A–P axis. One stimulation site in the SPL in Patient 2 was excluded from the analysis because of our interest in the frontal lobe. This sub-analysis revealed that the locations of the stimulation electrodes with a statistically significant increase in the No-Go errors were more anterior than those without No-Go error increase [Y coordinate in the MNI standard space: No-Go error increase group = 22.4 ± 2.1 mm (*n* = 3), No-Go error non-increase group = 13.2 ± 9.3 mm (*n* = 8)] (Fig. 3B), although the statistical test could not be performed because of the small sample size. Similarly, when focusing on the frontal lobe, the locations of language-related areas were more anterior than that of NMAs [Y coordinate in the MNI standard space: NMA = 4.5 ± 11.3 mm (*n* = 40), language-related area = 17.9 ± 7.6 mm (*n* = 24), *P* < 0.0001, d = 6.49, Wilcoxon rank sum test] (Fig. 2C). When restricting the analysis to the functional areas that overlapped with large No-Go ERP sites, the electrodes in the language-related areas were located more anteriorly than were the NMA electrodes [Y coordinate in the MNI standard space: NMA with large No-Go ERP = 9.0 ± 1.8 mm (*n* = 4), language-related area with large No-Go ERP = 14.0 ± 4.7 mm (*n* = 12)] (Fig. 2C), although the statistical test could not be performed because of the small sample size.

## Discussion

To summarize our results, (i) anatomically, No-Go-specific ERPs with relatively high amplitude (large No-Go ERPs) were observed predominantly in the secondary motor areas, which include the SMA proper, pre-SMA and lateral premotor areas (Fig. 2A). (ii) Functionally, large No-Go ERPs in the frontal lobe were located in or around the NMAs or language-related areas (Fig. 2). (iii) Stimulation prolonged the Go RT at most stimulation sites (66.7%) and increased the No-Go errors at some stimulation sites (25.0%, left pMFG and left pre-SMA) (Figs. 3 and 4). (iv) The stimulation sites selected for brief stimulation were most frequently labelled as ‘NMA’ (63.6%), followed by ‘language-related area’ (18.2%) in the ECS mapping (Table 1). (v) The stimulation sites where brief stimulation led to increased No-Go errors tended to be labelled as the ‘language-related area’ more frequently than as ‘NMA’ [*P* = 0.0833, Fisher’s exact test (two-sided)] (Table 2) and were located more anteriorly than those without No-Go error increase (Fig. 3B).

### Critical areas for response inhibition

The finding of the large No-Go ERPs in the secondary motor areas (Fig. 2A) is consistent with previous fMRI studies.^7^ We further observed the frequent overlap of sites with No-Go-specific ERPs, Go RT prolongation on brief stimulation, and NMA labelling in ECS mapping (Figs. 2 and 3, Tables 1 and 2). We assumed that Go RT prolongation and negative motor responses are caused by a deficit in the initiation and maintenance of well-planned action, respectively. Therefore, our results suggest a close relationship between response inhibition and action execution.

The anatomical locations of NMAs in the present study (Fig. 2B) were consistent with those described in the previous studies, showing that the NMAs were distributed throughout the precentral gyrus^17^ or the perirolandic area.^27^ The functional role of the NMAs remains unclear; however, some researchers have suggested that the NMAs engage in inhibitory control of action.^15^ The partial overlap of the locations of large No-Go ERPs and NMA observed in this study (Fig. 2, Table 2) suggests that some of the NMAs are associated with inhibitory control of action.

A No-Go error increase, the behavioural index of impaired inhibitory control, was caused by stimulation at some of the No-Go ERPs-positive areas, namely, the left pre-SMA and left pMFG (Figs. 3A and 4B). Thus, our results provide additional important evidence corroborating previous TMS studies suggesting that the pre-SMA and pMFG play an important role in response inhibition.^8,9^ Furthermore, we found a frequent overlap of the sites where brief stimulation increased the No-Go error with those labelled as language-related areas in the ECS mapping (Tables 1 and 2), suggesting a common mechanism underlying the deficit of response inhibition and language tasks. Previous studies have suggested that the PMd engages in making decisions about movement according to the prevailing rules,^23,28–30^ whereas the pre-SMA plays an important role in the contextual control of voluntary behaviour.^13,30^ Such flexible executive action control may be vital for success in both response inhibition and language tasks. Therefore, our results suggest that part of the pre-SMA and pMFG engage in the early phase of executive action control, such as action planning according to the prevailing rules and context.

In 5 out of 8 patients, the intracranial electrodes were implanted on the language-dominant hemisphere. It may mean that some components of ERPs reflected the linguistic processing necessary for stimulus-response translation, or that stimulation-induced behavioural changes derived from the disturbance of such linguistic processing. Therefore, it is interesting to evaluate the relationship of hemispheric laterality with ERPs or stimulation-induced behavioural changes in the future study.

Furthermore, we established that the language-related areas were more anterior in the frontal lobe than the NMAs (Fig. 2B and C). In addition, the stimulation electrodes at the sites with increased No-Go errors tended to be labelled as language-related area more frequently than as NMAs (Table 2), although statistically not significant.

Considering these results, we propose that the secondary motor areas play an important role in linking cognition to action with functional differentiation along the A–P axis. More anterior areas engage in the earlier phase of executive action control with a focus on action programming, such as response inhibition and action planning, whereas more posterior areas take part in the later phase of executive control with an emphasis on action execution, such as the initiation and maintenance of well-planned action. This notion is supported by the presence of a connectivity gradient between the lateral and medial motor cortices along the A–P axis, as studied using cortico-cortical evoked potentials.^24^

In the present study, we focused on frontal lobe electrodes. However, large No-Go ERP sites and NMAs were also observed in the parietal lobe, namely, the inferior parietal lobule (Fig. 2), suggestive of the involvement of the inferior parietal lobule in response inhibition. Recent studies using fMRI or TMS have shown the contribution of the parietal lobe, especially the intraparietal sulcus, in response inhibition.^31,32^ TMS over the intraparietal sulcus region prolonged the stop-signal RT, and a parcellation-based network analysis of resting-state fMRI showed a connection between the intraparietal sulcus and the inferior frontal cortex and pre-SMA.^32^ In the present study, as for parietal lobe, stimulation for the brief stimulation study was performed only at the SPL in Patient 2, not the inferior parietal lobule, resulting in no significant behavioural changes. Therefore, we could not provide strong evidence that the parietal lobe, including the inferior parietal lobule, actively engages in response inhibition.

On the other hand, in some patients, the brief stimulation at the left pMFG (Patient 2) and left pre-SMA (Patient 3) decreased the errors in the Go and No-Go trials, respectively (Figs. 3A, 4B, and C). The previous critical article argued that 50 Hz stimulation has excitatory or inhibitory effect remains unclear.^33^ It is assumed that the effects of the stimulation are mediated by disturbance (mixture of activation and inhibition) of the multiple regions connected to the stimulation site. We speculate that the variability of the brief stimulation results across patients may result from the difference in the degree of disturbance of the executive action control system. Our results suggest that the response inhibition function is attributable to multiple cortical regions. This multi-regional processing hypothesis is supported by a recent study that combined magnetoencephalography and TMS and showed simultaneous activity across the right inferior frontal gyrus and pre-SMA in response inhibition.^34^ In addition, some subcortical regions, especially the subthalamic nucleus, are also thought to play a critical role in response inhibition.^35^ We hypothesized that parallel and mutually interdependent processing by multiple cortical and subcortical regions might bring about the variability of the brief stimulation results in the present study.

### Clinical implications

A recent intraoperative brain mapping study showed that the dorsolateral prefrontal cortex, especially the posterior part of the middle frontal gyrus, plays a critical role in semantic cognition.^36^ In addition, the pre-SMA has been thought to be associated with speech initiation and verbal fluency through a recently identified monosynaptic connection with the lateral inferior frontal gyrus, namely, the frontal aslant tract.^37^ Our results further suggest that some of these language-related areas also engage upstream in the control of executive action, such as action planning, rather than in language function per se. To further delineate language-related areas, that is, differentiating them from the core language area, modification of the behavioural tasks would be beneficial for ECS mapping. For example, screening of hand or orobuccal apraxia during ECS prior to language function mapping would be useful.

In addition, the present study suggested that the combination of ECoG and ECS would be applicable for future advanced clinical ECS mapping to preserve higher brain function, such as response inhibition, in resective brain surgery.

### Limitations

The present study had several limitations. The number of subjects was relatively small (*n* = 8). We supposed our ECoG study with ECS had the advantage of combining the causal information from ECS with correlational information from ERP recording in each patient, providing higher specificity for mapping the cortical regions critical in response inhibition than the previous fMRI, TMS, or ECoG studies without ECS. However, investigation with a larger number of subjects would be needed in the future. The locations of the implanted electrodes varied among patients due to the clinical demands of presurgical evaluation. Because of the clinical purpose of ECS mapping, such as language function mapping, the majority of patients (five out of eight patients) had electrode coverage in the left hemisphere. In addition, we performed brief stimulation in the right hemisphere for only two patients, making it difficult to evaluate the laterality of the response inhibition function. In the present study, we could not standardize the current intensity of the stimulation, which ranged between 4 and 8 mA, due to seizure induction risk (patient 3) or negative motor response (patient 8). We could not completely exclude arbitrariness in the selection of stimulation sites in the brief stimulation study. However, we retrospectively confirmed that most stimulation sites showed No-Go-specific ERPs, suggesting that our choice of the stimulation sites was generally reasonable. Since the number of the patients, the stimulation sites, or the variability of the stimulation intensity was small, we could not analyze the interaction effects of stimulation site by stimulation intensity, or the main effect of stimulation site on Go RT and No-Go errors. Since all patients had frontal lobe epilepsy, we could not completely exclude the possibility that the results of the present study entirely reflected a pathological response due to aberrant epileptic networks, especially in the patients who had epileptic focus at or around the areas of interest of the present study, namely, pre-SMA or lateral premotor areas. In addition, the background of the patients varied regarding cognitive function, seizure focus, underlying pathology (Supplementary Table 1), electrode locations, number of electrodes, and antiepileptic drugs, leading to potential biases. We attempted to resolve these potential biases by analyzing patients as a group, not individually. The convergent results in the MNI standard space, despite the variable patient background, suggest that the results of the present study reflect normal brain function.

## Conclusions

By integrating the methods of different modality, namely, ERPs combined with brief stimulation and clinical ECS mapping, we conducted a novel neuroscientific approach, providing direct evidence that the secondary motor areas, especially the pre-SMA and pMFG, have an important role in response inhibition.

## Supplementary Material

fcac204_Supplementary_Data

## Abbreviations

A–P =: anterior–posterior
ECoG =: electrocorticography
ECS =: electrical cortical stimulation
ERPs =: event-related potentials
fMRI =: functional MRI
MNI =: Montreal Neurological Institute
NMAs =: negative motor areas
PMd =: dorsal premotor area
pMFG =: posterior middle frontal gyrus
PMv =: ventral premotor area
pre-SMA =: pre-supplementary motor area
RT =: reaction time
SD =: standard deviation
SPL =: superior parietal lobule
TMS =: transcranial magnetic stimulation
VAC =: vertical anterior commissure
