# Supplementary material for: Secondary motor areas for response inhibition: an epicortical recording and stimulation study
Source: Brain Commun. 2022 Aug 4;4(4):fcac204. doi: 10.1093/braincomms/fcac204 (PMC9380994; doi:10.1093/braincomms/fcac204)
Supplement: fcac204_Supplementary_Data [file fcac204_supplementary_data.pdf]

Supplementary Table 1: Patients' demographics and clinical information

Patient No., age/gender, handedness	Electrode implantion side	Age of seizure onset	Ictal onset area	Pathology
1. 39M, R	L	8	Pre-SMA, PMd, DLPFC	FCD IIB
2. 45M, L	L	15	SMA, PMd	FCD IA
3. 29M, L	R	18	Pre-SMA, PMd, PMv	FCD IA
4. 16F, R	L	14	PMv	DNT
5. 39F, R	L	12	Pre-SMA, PMd	FCD IIB
6. 23F, L	L	13	Ventral postcentral & precentral gyrus	FCD IIB
7. 20M, R	R	11	Prefrontal area	FCD IIB
8. 24F, R	R	12	Insular, frontal operculum	FCD IIA<br>HS

L: left, R: right, SPS: simple partial seizure, DNT: dysmorphic neuroepithelial tumor, FCD: focal cortical dysplasia (Palmini classification), HS: Hippocampal sclerosis, SMA: supplementary motor area, Pre-SMA: pre-supplementary motor area, PMd: dorsal premotor area, PMv: ventral premotor area, DLPFC: dorsolateral prefrontal cortex.

Supplementary Table 2: The behavioral results of the Go/No-Go task for ERP recording

Patient No.	Go Reaction time (ms)	No-Go Error (%)	Go error (%)
1	350.5 ± 64.9	1.4	0.9
2	404.8 ± 86.5	3.1	4.9
3	321.3 ± 81.1	22.9	3.1
4	337.5 ± 50.8	2.1	1.7
5	373.8 ± 67.6	6.7	1.1
6	415.2 ± 155.1	1.1	2.9
7	370.4 ± 65.5	7.3	1.7
8	355.6 ± 95.2	2.1	1.1
All	365.3 ± 93.5	5.8 ± 7.3	2.2 ± 1.4

Supplementary Figure 1. Electrode placement in patients.

Electrode placement in A) subdural electrode implantation cases (patients 1–6) and B) depth electrode implantation cases (patients 7 and 8). Upper, middle, and lower panels: three-dimensional view from the medial (upside down), dorsal, and lateral side, respectively. The colored dots indicate the locations of the implanted electrodes in the MNI standard space. In panel B, note that most electrodes were implanted deep inside the brain, and few electrodes were located on the cortical surface. * The electrodes in patients 3, 7, and 8 are swapped from right to left side for display purposes. A: Anterior, P: Posterior.

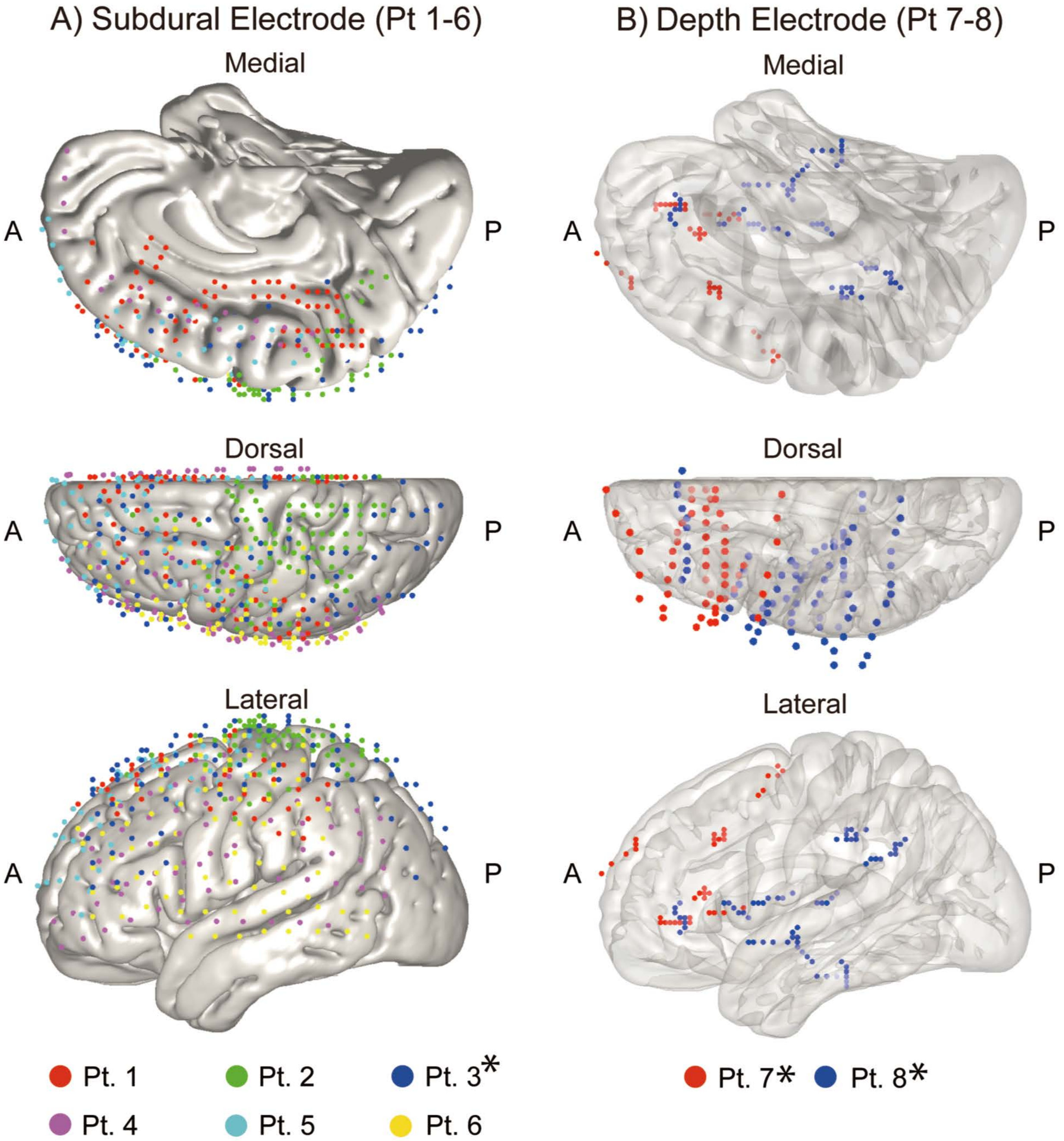

A) Subdural Electrode (Pt 1-6)
B) Depth Electrode (Pt 7-8)
Medial
Dorsal
Lateral
A
P
Pt. 1
Pt. 2
Pt. 3*
Pt. 4
Pt. 5
Pt. 6
Pt. 7*
Pt. 8*

Supplementary Figure 2. Positive rate (%) of the large No-Go ERP site, NMAs, language-related areas, and their overlap.

The positive rate (%) of the electrodes (large No-Go ERP sites, NMAs, language-related areas, NMA with large No-Go ERP, and language-related area with large No-Go ERP) are shown as box-plots. The positive rate was not different between NMA and language-related areas ($p = 0.7513$, Wilcoxon rank sum test) and between NMA with large No-Go ERP and language-related areas with large No-Go ERP ($p = 0.6516$, Wilcoxon rank sum test).

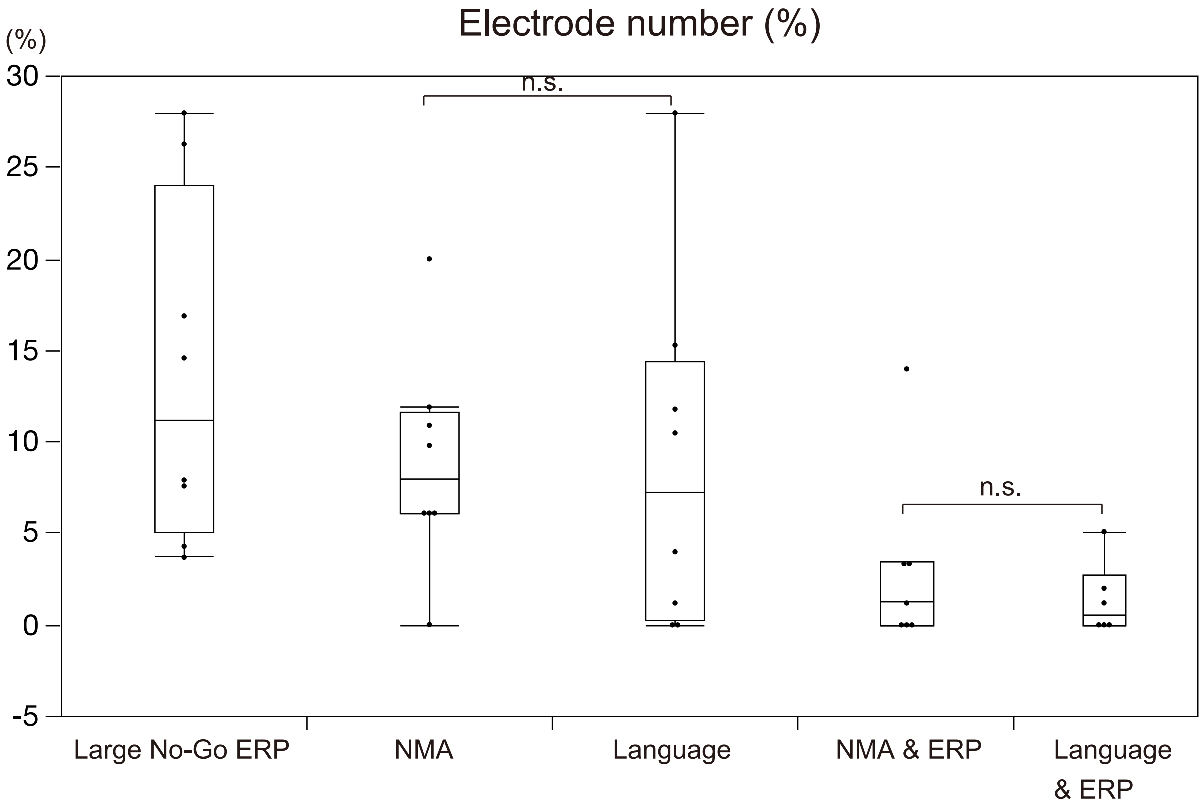

Electrode number (%)
(%)
30
25
20
15
10
5
0
-5
n.s.
n.s.
Large No-Go ERP
NMA
Language
NMA & ERP
Language & ERP
